# Clinical significance of circulatory chlamydial heat shock protein-60 antibodies in spondyloarthropathy patients

**DOI:** 10.1186/1471-2334-14-S3-P59

**Published:** 2014-05-27

**Authors:** Praveen Kumar, Darshan Singh Bhakuni, Sangita Rastogi

**Affiliations:** 1 Microbiology Laboratory, National Institute of Pathology (ICMR), Safdarjung Hospital campus, New Delhi, India; 2 Department of Rheumatology and Clinical Immunology, Army Hospital (Research & Referral), New Delhi, India

## Background

*Chlamydia trachomatis* is a multifaceted, asymptomatic, most prevalent sexually transmitted pathogen worldwide. Due to lack of specific diagnostic methods for *C. trachomatis* in Spondyloarthropathy (SpA) patients, this pathogen is under diagnosed and under estimated in various countries including India. We aimed to estimate circulatory antibodies to chlamydial heat shock protein-60 (chsp60) IgG/ anti *C. trachomatis* IgA and evaluate their clinical significance in SpA patients.

## Methods

In this case control study, serum from 60 arthritic patients comprising of Reactive Arthritis (ReA), undifferentiated SpA, Rheumatoid Arthritis (RA) and Osteoarthritis (OA) was collected for detection of anti chsp60 IgG and anti *C. trachomatis* IgA antibodies by using commercial ELISA kits. Cut-off indices (COI) of infected SpA patients were correlated with clinical data.

## Results

Overall, 12/ 30 (40.0%) SpA patients, viz.: ReA/uSpA, were found positive for either circulatory chsp60 IgG or anti *C. trachomatis* IgA antibody (*p* value < 0.009 versus RA/ OA). 23.3% (7/ 30) SpA patients and 3.3 % (1/30) RA/OA control patients were found to be positive for circulatory chsp60 IgG antibodies. 16.6% (5/ 30) patients found positive for anti *C. trachomatis* IgA antibodies; however, none of the controls was infected. COI, age and disease duration in chsp60-positive SpA patients showed significant difference in median values (Kruskal-Wallis test; *p* value < 0.0001) while there was no such relation to anti *C. trachomatis* IgA.

## Conclusion

Overall findings indicate an active/chronic *C. trachomatis* infection in SpA patients. The study also suggests usefulness of chsp60 serology to facilitate better clinical management of patients.

